# Supplementary material for: Disruption of Cancer Metabolic SREBP1/miR-142-5p Suppresses Epithelial–Mesenchymal Transition and Stemness in Esophageal Carcinoma
Source: Cells. 2019 Dec 18;9(1):7. doi: 10.3390/cells9010007 (PMC7016574; doi:10.3390/cells9010007)
Supplement: Supplementary file 1 [file cells-09-00007-s001.pdf]

**SUPPLEMENTARY INFORMATION**

# Disruption of cancer metabolism SREBP1/miR-142-5p circuit suppresses epithelial-mesenchyaml transition and stemness in esophageal carcinoma

Chih-Ming Huang 1#, Chin-Sheng Huang 2,3#, Tung-Nien Hsu2,3, Mao-Suan Huang2,3, Iat-Hang Fong4,5, Wei-Hwa Lee4,5*, Shao-Cheng Liu6*

1 Department of Otolaryngology, Taitung Mackay Memorial Hospital, Taitung City 950, Taiwan;
2 Division of Oral and Maxillofacial Surgery, Department of Dentistry, Taipei Medical University - Shuang Ho Hospital, New Taipei City 235, Taiwan;
3 School of Dentistry, College of Oral Medicine, Taipei Medical University, Taipei City 110, Taiwan;
4Department of Medical Research & Education, Taipei Medical University - Shuang Ho Hospital, New Taipei City, 235, Taiwan
5Department of Pathology, Taipei Medical University-Shuang Ho Hospital, New Taipei City, Taiwan
6 Department of Otolaryngology-Head and Neck Surgery, Tri-Service General Hospital, National Defense Medical Center, Taipei City, 114, Taiwan

#Co-first authors.
* Corresponding Author
Wei-Hwa Lee, MD., PhD. Professor
Department of Pathology, Taipei Medical University-Shuang Ho Hospital, New Taipei City, Taiwan.
Phone: +886-2-2490088 ext. 8742; FAX: 886-2-2248-0900; E-mail: whlpath97616@s.tmu.edu.tw

Shao-Cheng Liu, MD., Ph.D. Associate Professor
Department of Otolaryngology-Head and Neck Surgery, Tri-Service General Hospital, National Defense Medical Center, Taipei City, 114, Taiwan
Phone: +886-2-87927192. FAX: 886-2-87927193
E-mail: m871435@ndmctsgh.edu.tw

**Supplementary Table S1**

| No. | | Target | Dilution | Source | |
|---|---|---|---|---|---|
| 1 | Western blots | SREBP-1 | 1:1000 | ab191857 | abcam |
| 2 | | ZEB1 | 1:2000 | ab228986 | abcam |
| 3 | | Vimentin | 1:1000 | #5741 | cellsignaling |
| 4 | | E-Cadherin | 1:1000 | 20874-1-AP | PROTEINTECH |
| 5 | | GAPDH | 1:10000 | 10494-1-AP | PROTEINTECH |
| 6 | Immunohistochemistry | SREBP-1 | 1:100 | ab28481 | abcam |
| 7 | | ZEB1 | 1:100 | ab228986 | abcam |
| 8 | | Vimentin | 1:200 | ab92547 | abcam |
| 9 | | E-Cadherin | 1:500 | 20874-1-AP | PROTEINTECH |
| 10 | | Ki-67 | 1:100 | MA5-14520 | ThermoFsher |

**Supplementary Figure S1**

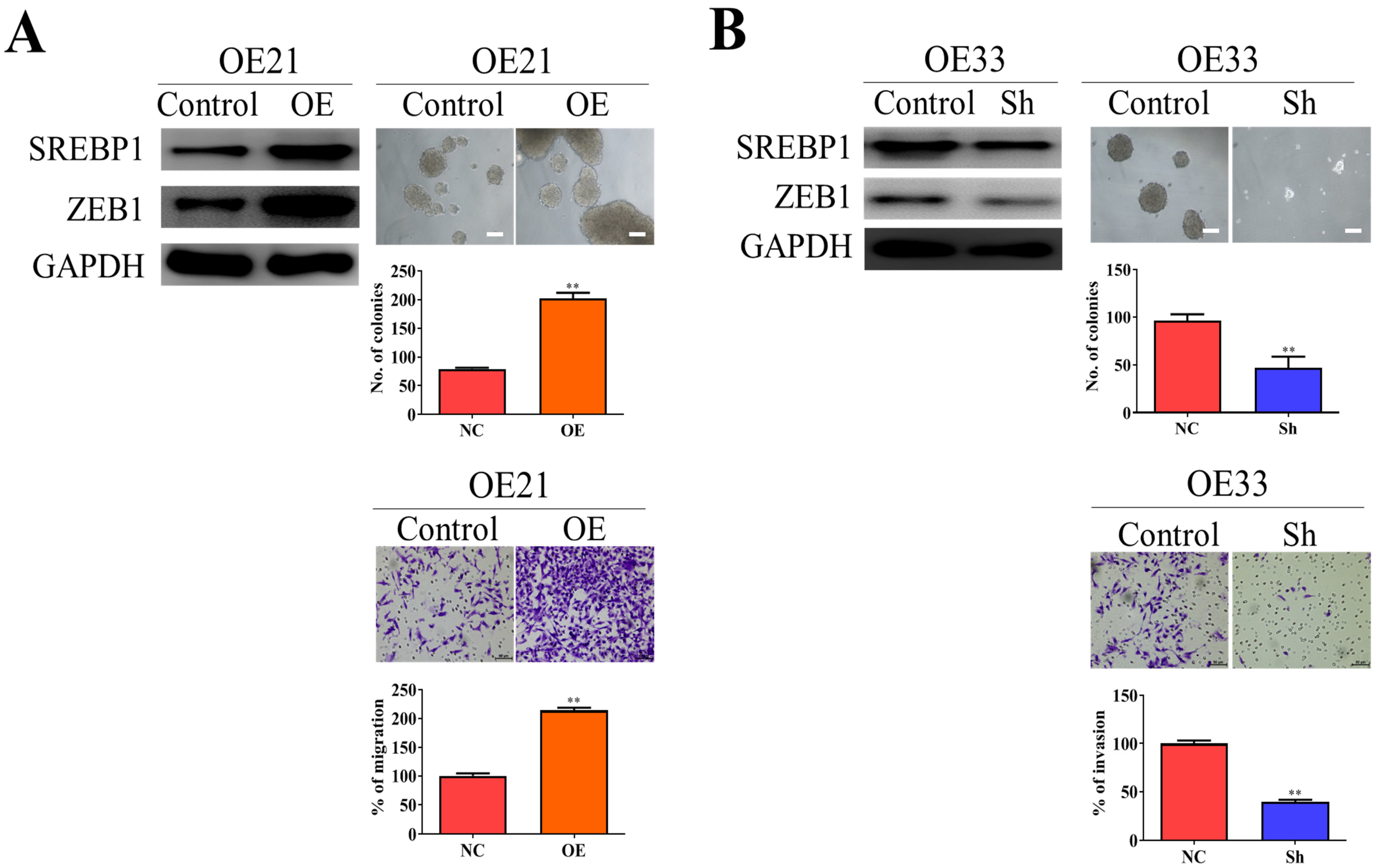


**Supplementary Figure S1.** Overexpression (OE) and silencing (Sh) of SREBP1 in OE21 and OE33 cells. (A) SREBP1-overexpressing OE21 showed increased ZEB1 expression accompanied with enhanced tumor sphere forming (upper panel) and invasive potential (lower panel). (B) SREBP1-silenced OE33 cells showed the opposite effects.

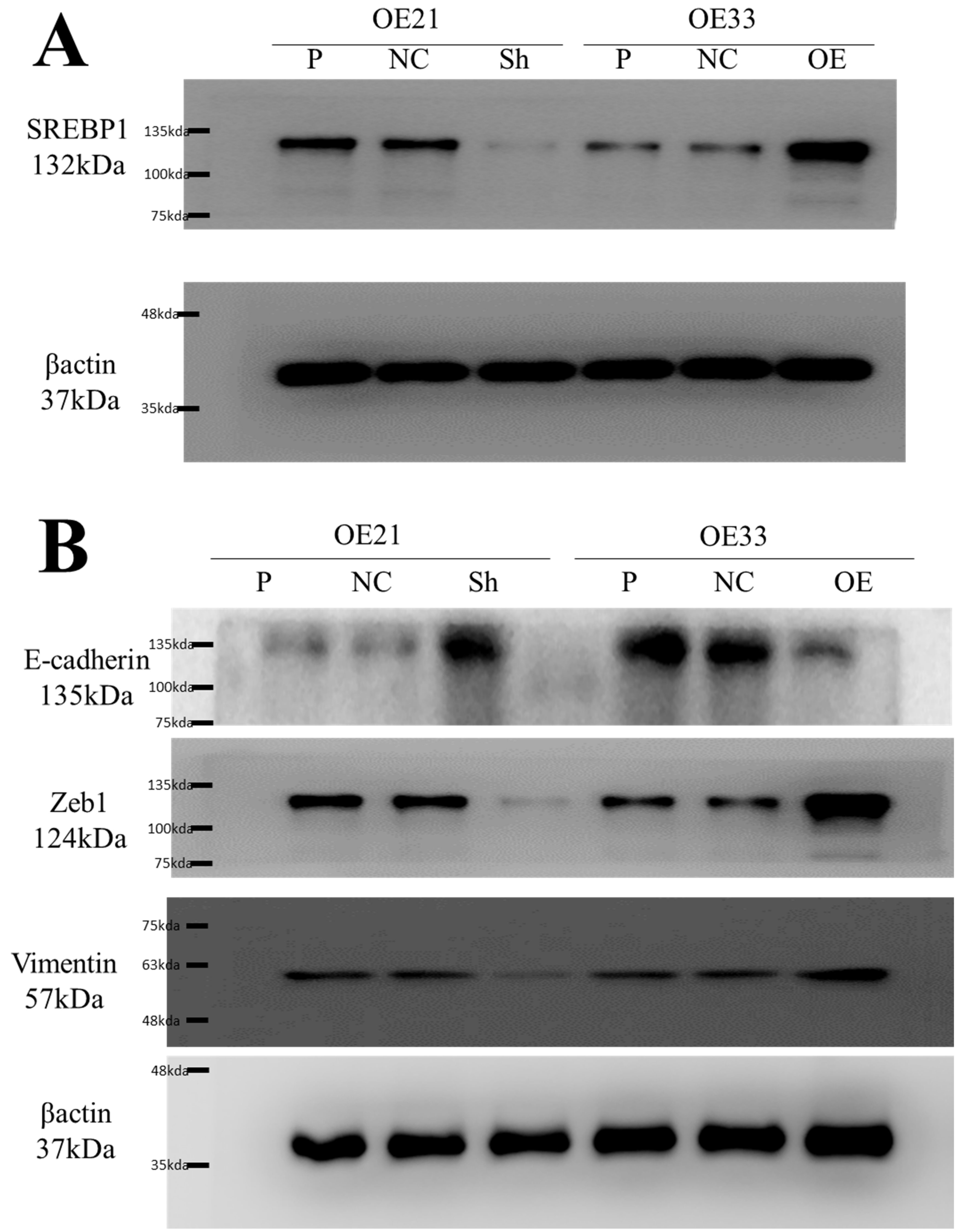


**Supplementary Figure S2.** Full-size blots of Figure 2

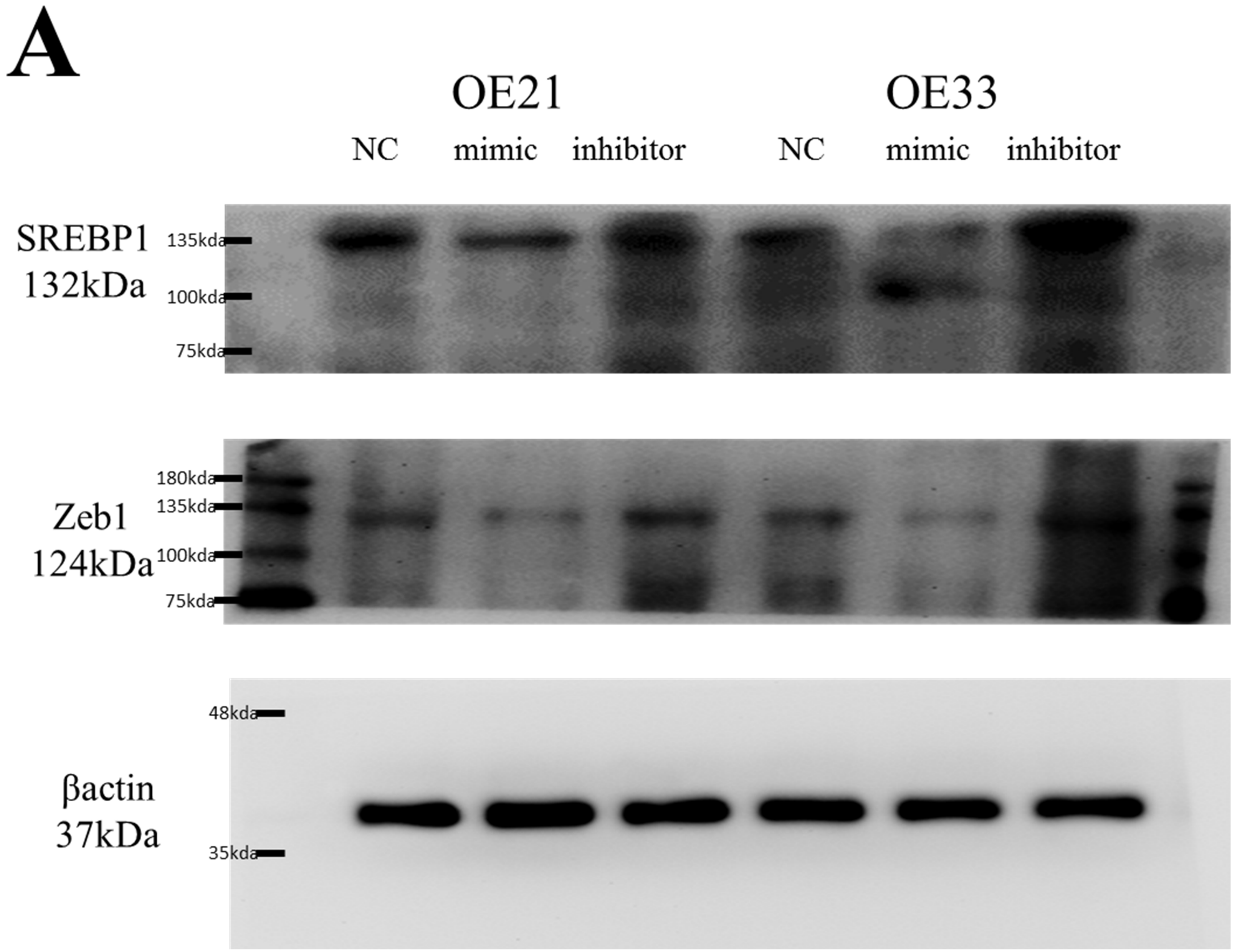


**Supplementary Figure S3.** Full-size blots of Figure 3C

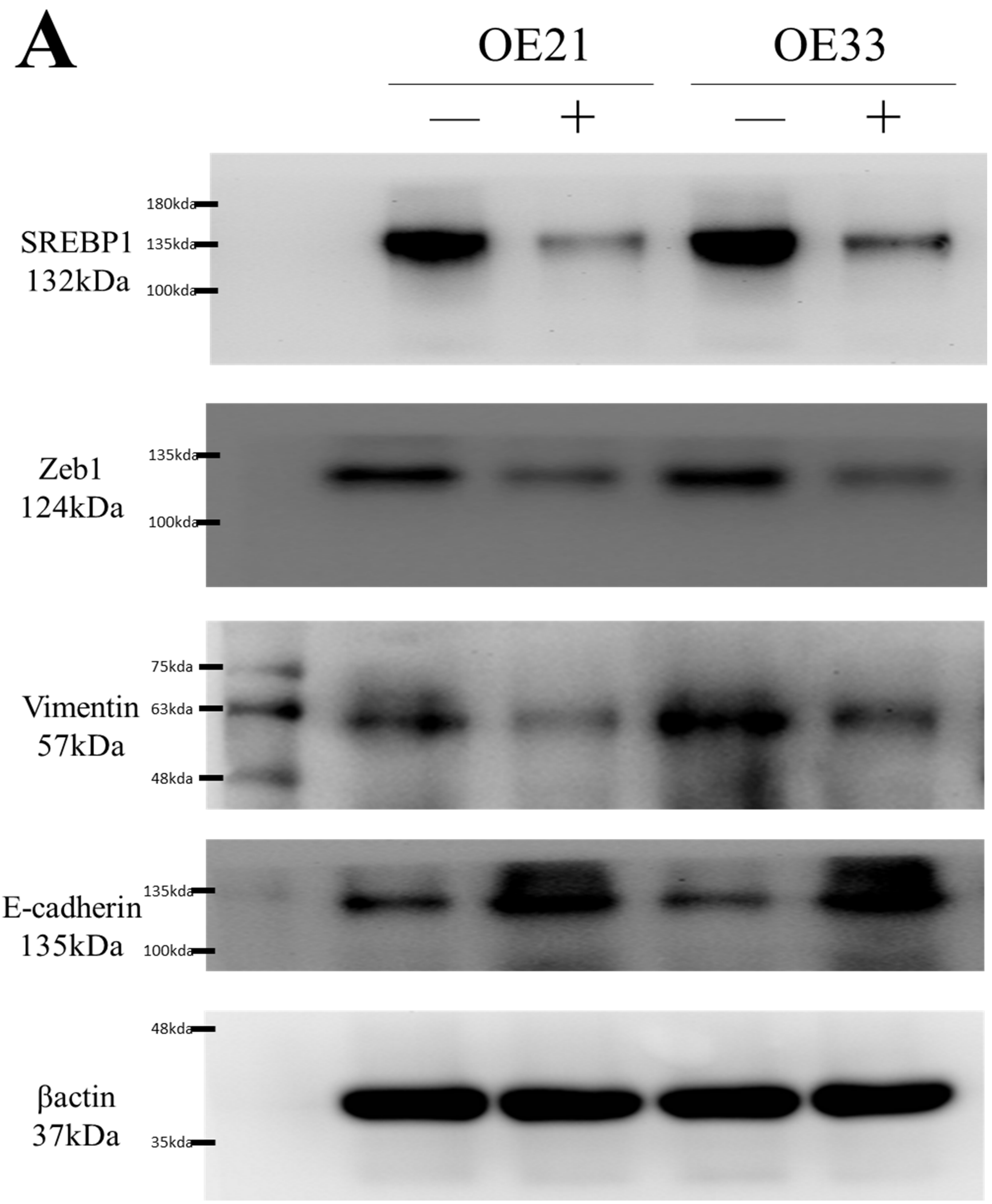


**Supplementary Figure S4.** Full-size blots of Figure 4C

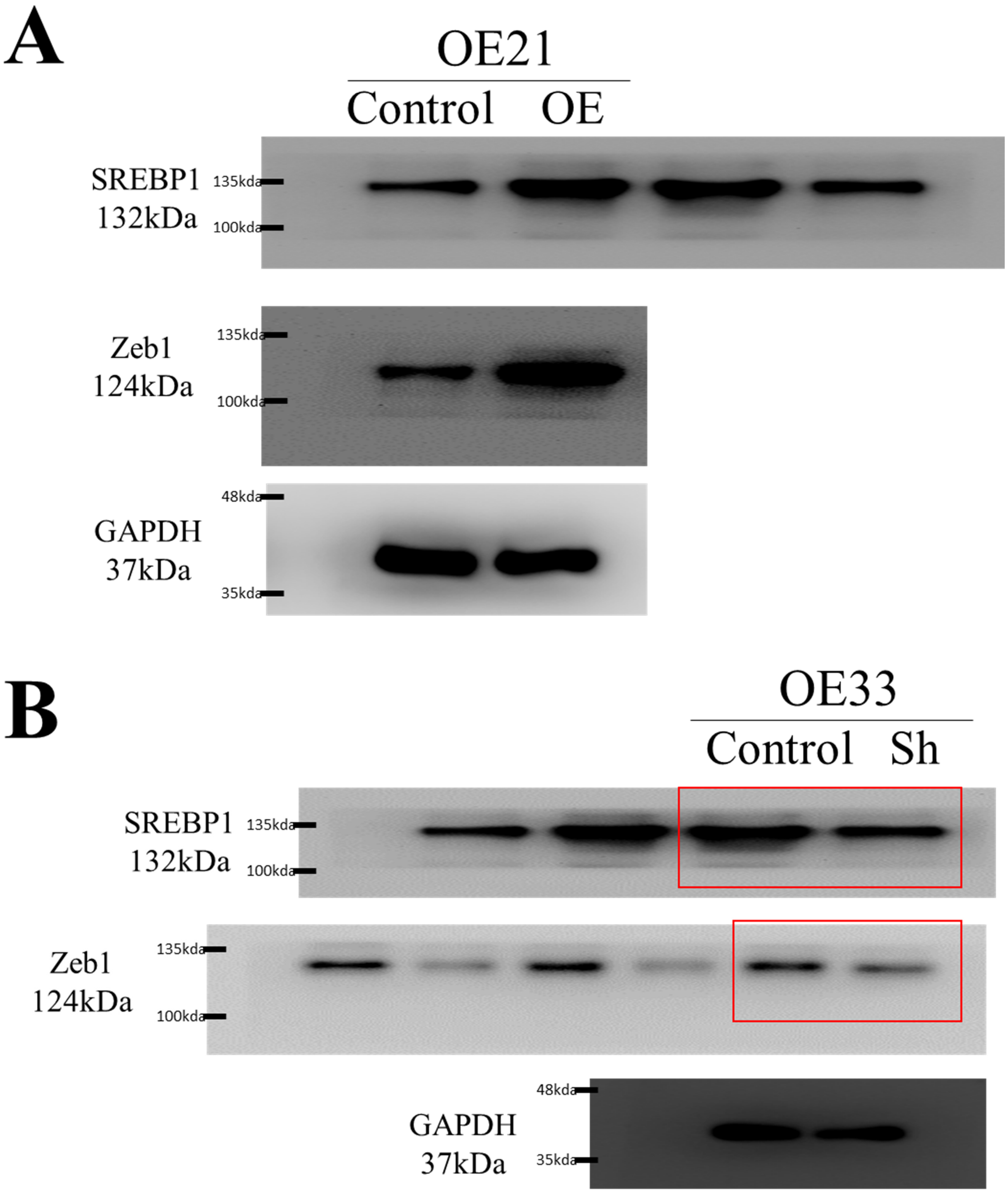


**Supplementary Figure S5.** Full-size blots of Supplementary Figure S1
